# Glomerular filtration rate prior to high-dose melphalan 200 mg/m^2^ as a surrogate marker of outcome in patients with myeloma

**DOI:** 10.1054/bjoc.2000.1928

**Published:** 2001-08

**Authors:** B Sirohi, R Powles, S Kulkarni, C Rudin, R Saso, A Rigg, C Horton, S Singhal, J Mehta, J Treleaven

**Affiliations:** Leukaemia and Myeloma Units, Royal Marsden NHS Trust, Surrey, UK

**Keywords:** myeloma, glomerular filtration rate, morbidity, outcome

## Abstract

We correlated age and body surface area corrected glomerular filtration rate (GFR) at the time of high-dose melphalan (HDM) administration with treatment-related toxicity (TT), time to disease progression and survival. Between 8/85 and 6/98, 144 newly diagnosed myeloma patients with a median age of 53 years (range, 31–72) received infusional chemotherapy with vincristine, doxorubicin and methylprednisolone, with/without cyclophosphamide or verapamil, followed by HDM 200 mg/m^2^and stem cell rescue. An additional patient received HDM at diagnosis. GFR was below normal in 38 patients (26%). At presentation, patients with low GFR at the time of HDM had higher serum creatinine, β_2_M, stage III disease, calcium and Bence–Jones proteinuria. Toxic deaths post-HDM were similar in both groups (2/38 (5%) vs. 4/107 (4%)), though patients with low GFR had more oral mucositis (*P*< 0.0001), diarrhoea (*P* = 0.005) and infections (*P* = 0.04). The response and relapse rates of the 2 groups were not substantially different, but the median overall survival (OS) was significantly shorter in patients with low GFR (5.1 vs 7.5 years, *P* = 0.015). Multivariate analysis showed that a normal GFR and being in CR at the time of HDM were predictive of longer OS. We conclude that in context of high-dose chemotherapy for myeloma, dose of melphalan should not be modified in patients with low GFR and that early intensive treatment at relapse may improve results in patients with abnormal renal function. © 2001 Cancer Research Campaign http://www.bjcancer.com


*British Journal of Cancer* (2001) **85**(3), 325–332
© 2001 Cancer Research Campaign
doi: 10.1054/ bjoc.2000.1928, available online at http://www.idealibrary.com on IDEAL
http://www.bjcancer.com

### Glomerular filtration rate prior to high-dose melphalan 200 mg/m² as a surrogate marker of outcome in patients with myeloma

**B Sirohi, R Powles, S Kulkarni, C Rudin, R Saso, A Rigg, C Horton, S Singhal[1], J Mehta[1] and J Treleaven**

Leukaemia and Myeloma Units, Royal Marsden NHS Trust, Surrey, UK

**Summary** We correlated age and body surface area corrected glomerular filtration rate (GFR) at the time of high-dose melphalan (HDM) administration with treatment-related toxicity (TT), time to disease progression and survival. Between 8/85 and 6/98, 144 newly diagnosed myeloma patients with a median age of 53 years (range, 31–72) received infusional chemotherapy with vincristine, doxorubicin and methylprednisolone, with/without cyclophosphamide or verapamil, followed by HDM 200 mg/m² and stem cell rescue. An additional patient received HDM at diagnosis. GFR was below normal in 38 patients (26%). At presentation, patients with low GFR at the time of HDM had higher serum creatinine, $\beta_2$M, stage III disease, calcium and Bence–Jones proteinuria. Toxic deaths post-HDM were similar in both groups (2/38 (5%) vs. 4/107 (4%)), though patients with low GFR had more oral mucositis ($P < 0.0001$), diarrhoea ($P = 0.005$) and infections ($P = 0.04$). The response and relapse rates of the 2 groups were not substantially different, but the median overall survival (OS) was significantly shorter in patients with low GFR (5.1 vs 7.5 years, $P = 0.015$). Multivariate analysis showed that a normal GFR and being in CR at the time of HDM were predictive of longer OS. We conclude that in context of high-dose chemotherapy for myeloma, dose of melphalan should not be modified in patients with low GFR and that early intensive treatment at relapse may improve results in patients with abnormal renal function.
© 2001 Cancer Research Campaign http://www.bjcancer.com

**Keywords**: myeloma; glomerular filtration rate; morbidity; outcome

Renal function is one of the most important prognostic factors in patients with multiple myeloma (Durie and Salmon, 1975). During the last 20 years myeloma treatment has changed substantially and now typically constitutes initial courses of infusional chemotherapy (IC) with VAMP/C-VAMP/VAD (vincristine, doxorubicin, and methylprednisolone or dexamethasone with or without cyclophosphamide) followed in approximately 70% of patients by consolidation with HDM and autologous stem cell rescue with either cryopreserved marrow or peripheral blood stem cells (Powles et al, 1997; Barlogie et al, 1999). Autotransplantation is now considered for myeloma patients up to the age of 75 (Siegel et al, 2000, Sirohi et al, 2000) which implies that the majority of patients with myeloma may benefit from this treatment. Patients with renal dysfunction, however, are frequently excluded from high dose protocols, even though the case for this practice has been weakened since the French and Little Rock groups have shown that renal insuffciency may not be a valid criterion for exclusion from high-dose therapy programs (Kergueris et al, 1994; Tricot et al, 1996).

We have previously reported that some patients who present with renal dysfunction recover normal renal function after IC, whereas others go on to develop renal dysfunction during IC (Sirohi et al, 1999a). The purpose of the present observational study was to assess whether renal function just prior to high-dose treatment is of prognostic value with respect to treatment-related toxicity, disease progression and survival. As measure of renal function we used the glomerular filtration rate (GFR) corrected for age, determined not more than 30 days prior to the HDM. All the 145 patients described here were treated uniformly with HDM 200 mg m$^{-2}$.

*Received 25 October 2000*
*Revised 17 April 2001*
*Accepted 17 April 2001*

*Correspondence to:* R Powles

### PATIENTS AND METHODS

### Patients

From a prospectively maintained database, 145 newly diagnosed patients with multiple myeloma who received HDM 200 mg m$^{-2}$ as consolidation therapy were analysed. They presented to our unit between August 1985 and June 1998. Written informed consent to treatment was obtained according to institutional guidelines.

### Therapy

*Infusional chemotherapy (IC)*
Prior to the HDM, 144 patients were treated with infusional chemotherapy (IC) with VAMP ($n$ = 27), C-VAMP ($n$ = 97) or V-C-VAMP ($n$ = 20) (vincristine, doxorubicin, methylprednisolone with or without cyclophosphamide or verapamil; Forgeson et al, 1988; Raje et al, 1997a; Gore et al, 1988). All cycles were repeated 3 weekly until maximum response and followed by an additional cycle. One patient received HDM with an autologous peripheral stem cell graft at diagnosis.

Current address: [1]Division of Transplantation Medicine, South Carolina Cancer Center and Richland Memorial Hospital, Seven Richland Medical Park, Columbia, SC 29203

### *Patient evaluation pre HDM*

All patients who were planned to receive HDM 6 weeks after the last IC cycle had their GFR measured within 30 days before the HDM and this was the most recent value. GFR was assessed by a standard method using $^{51}$Cr EDTA as tracer (Brochner-Morteson, 1978) and corrected for body surface area (BSA) according to the formula Corrected GFR = Measured GFR × 1.73 / BSA.

Age-adjusted normal ranges were taken as:

20–39 years: 87–135 ml min$^{-1}$ 1.73 m$^{-2}$
40–49 years: 80–128 ml min$^{-1}$ 1.73 m$^{-2}$
50–59 years: 75–124 ml min$^{-1}$ 1.73 m$^{-2}$
< 60 years: 58–106 ml min$^{-1}$ 1.73 m$^{-2}$

GFR was not routinely measured post HDM unless the patients received a second course of high-dose therapy.

A full blood count, clotting screen, serum biochemistry, paraprotein, immunoglobulin quantitation, $\beta_2$ M, urinary Bence–Jones protein, bone marrow aspirate and trephine biopsy, chest X-ray, electrocardiogram and, if indicated, MUGA scan, were carried out just before the HDM.

### *High-dose treatment*

Since the publication of the reports by the French and Little Rock groups (Kergueris et al, 1994; Tricot et al, 1996) and after approval by the institutional review board, HDM has been included in the core care plan for patients with myeloma and renal impairment. All patients received melphalan at a dose of 200 mg m$^{-2}$ on day –1 as a bolus injection. Adequate hydration was ensured to maintain a urine output of 20 ml min$^{-1}$ during the first hour post high dose and 500 ml h$^{-1}$ in the subsequent 2 hours. Patients received autologous bone marrow ($n$ = 69) until November 1992 (Cunningham et al, 1994). The marrow was harvested 6 weeks after the last chemotherapy cycle. The target mononuclear cell count was 2 to 5 × 10$^{8}$ cells kg$^{-1}$ of body weight. From December 1992 onwards peripheral blood stem cell transplants ($n$ = 76) were used. The G-CSF mobilization procedure employed has been previously described (Powles et al, 1997; Raje et al, 1997b). The stem cells were infused 24 hours after the melphalan.

All patients were admitted to a 4-bedded open ward on the first day of conditioning and were nursed without barrier nursing. The Hickman lines inserted for IC were removed before HDM and all the patients received anti-pneumocystis carinii prophylaxis in the form of nebulized Pentamidine 300 mg once a month (starting on day –1). Packed red cells and platelets were transfused to keep the Hb> 9 gm dl$^{-1}$ and platelets > 10 × 10$^{9}$ l$^{-1}$ (in afebrile patients, > 20 × 10$^{9}$ l$^{-1}$ in febrile patients). Fluid and electrolyte balance was strictly maintained throughout the period of hospitalization. Gut sterilization was not used. Patients were commenced on empirical antibiotic therapy according to the hospital guidelines if pyrexia > 38˚C occurred. Mouth care included chlorhexidine mouth washes and nystatin suspension or amphotericin lozenges. A normal diet was encouraged. None of the patients required renal replacement therapy during the autotransplantation.

### *Maintenance therapy with interferon*

From July 1988 onwards, patients were started on maintenance treatment with interferon alpha, (3 megaunits m$^{-2}$ 3 times a week s/c) (Schering-Plough, Welwyn Garden City, Herts) following their recovery from HDM. Initially this took place as part of a randomized trial, but subsequently interferon maintenance was instituted in all patients as soon as the WBC count reached 2 × 10$^{9}$ l$^{-1}$ and the platelet count 50 × 10$^{9}$ l$^{-1}$. The dose of interferon was reduced or stopped according to the full blood count or other toxic manifestations (Cunningham et al, 1998).

### *Evaluation of toxicity*

Toxicity was assessed according to the WHO criteria (Miller et al, 1981). The variables analysed for TT were recovery of neutrophils to > 0.5 × 10$^{9}$ l$^{-1}$, recovery of platelets to > 50 × 10$^{9}$ l$^{-1}$, days of hospitalization, incidence of serum creatinine being > 177 μmol l$^{-1}$ (corresponding to stage B Durie–Salmon) during hospitalization, mucositis, diarrhoea, hepatic toxicity (bilirubin, transaminases, alkaline phosphatase) and microbiologically documented infection (DI). WHO grade III infections (moderate) are defined as febrile neutropenia with pyrexia > 40˚C unresponsive to antibiotic and antifungal therapy. WHO grade IV infections (severe) are neutropenic fevers associated with hypotension. Toxicity was attributed to HDM if it occurred during the patient's hospitalization for the transplant or the first 60 days following the transplant.

Transplant-related death was defined as death occurring during the first 60 days post autograft not related to disease relapse.

### Evaluation of response

The response in this group of patients has been evaluated pre-HDM as well as 3 and 6 months post HDM. Our response criteria and definition of complete remission (CR) have been described earlier by Gore et al (1989). Briefly, four criteria needed to be met together for a patient to be regarded as having achieved CR: (1) no measurable paraprotein on scanning densitometry of serum proteins separated electrophoretically on a cellulose acetate membrane and stained with ponceau S; (2) no detectable urinary Bence–Jones protein on electrophoresis of neat urine stained with colloidal gold; (3) no more than 5% plasma cells of normal morphology on the bone marrow aspirate and a normal bone marrow trephine biopsy; (4) stable skeletal appearances; (5) criteria 1–4 fulfilled for at least 3 consecutive months. Patients were regarded as having achieved a partial response (PR) if there was a 50% decrease in measurable paraprotein (IgG or IgA myeloma) or bone marrow infiltration (in the case of non-secretory or Bence–Jones myelomas) which was sustained for at least a month or more. Relapse was defined as either the reappearance of the paraprotein on 2 samples taken one month apart (or a 25% increase in paraprotein for patients in PR), bone marrow infiltration with more than 5% abnormal plasma cells, or development of new osteolytic lesions (or progression of old lesions). No response (NR) was considered to have occurred if patients failed to achieve a CR or a PR or had progression of disease (PD). Patients were considered to be in continued complete remission (CCR) or continued partial remission (CPR) if the CR/PR status attained after the induction infusional chemotherapy (IC) continued post HDM. Maximum response to IC was defined as either attainment of CR or a plateau phase for patients in PR (maximum reduction of the myeloma response criteria parameters). Patients were restaged on day 15 of the second and the fourth cycle of IC and if they fulfilled the above mentioned response criteria for restaging without the proviso of 3 months duration, they were said to have attained CR, PR or NR. The entry into the prospectively maintained database was done after the three months duration criterion was fulfilled and this was used for analysis.

*© 2001 Cancer Research Campaign*

### Statistical analysis

Patient characteristics were obtained at presentation and at high-dose therapy. Values of the characteristics were compared between groups of patients using non-parametric methods (Mann–Whitney or Kruskal–Wallis) for continuous numerical data or the Chi square test for categorical data. All probabilities were 2-tailed.

Outcome from the start of HDM was examined using overall survival (OS) and event-free survival (EFS) plots constructed via the Kaplan–Meier method (1952). Comparisons between such plots for different patient groups representing different categories of a given characteristic (i.e. univariate analysis) were compared via the log rank test (Peto et al, 1977). The event-points for the survival curves were death due to any cause for OS and relapse/death for EFS.

The categories chosen to represent the univariate influence of the continuous variables were based on partition values derived from either (a) median values or (b) single or multi-partition values chosen for optimum influence. These 2 types of partitioning (optimal cut-off) were also used for the multivariate analysis. The optimum values for (b) were found by using recursive partitioning which compared *P* values, produced via log-rank comparisons on Kaplan–Meier plots, with different partitioning values for the variable being studied (LeBlanc and Crowley 1993).

Multivariate analyses of the relationship between outcome and several variables were performed using forward stepwise regression by the Cox method (Cox, 1972; BMDP software package 1992). The forward stepwise method allows all variables to be considered for inclusion in the model but, in each step, selects the single variable that produces the largest, significant improvement of fit of the model (i.e. has the lowest P value). Successive steps of single selections from the pool of remaining variables are carried out until the improvement in *P* value is no longer significant (defined as $P > 0.05$). This procedure is preferable to the pre-selection of eligible variables on the basis of univariate *P* values because it allows for interrelationships between variables that may change their significance as the model develops.

The pre-autograft variables chosen to be included in the multivariate analysis were previously found to influence prognosis in patients with myeloma. They were age, serum calcium, $\beta_2$M, serum albumin, Hb, immunologic subtype of myeloma (IgG vs IgA vs light-chain/IgM/IgD/non-secretory), presence or absence of Bence–Jones proteinuria, type of light chain (kappa vs lambda), GFR (model run for GFR as dichotomous N/L value and as a continuous variable), type of IC (VAMP vs. CVAMP/V-CVAMP), type of autograft (marrow vs PBSC), Durie-Salmon stage of disease (I/II vs III), number of courses of IC, and response to IC (CR vs PR vs NR; (Merlini et al 1980; MRC 1980; Vesole et al, 1996; Sirohi et al, 1999b)). Because the addition of cyclophosphamide to IC and the number of courses of IC have an impact on CR, these variables were also included in the analyses (Raje et al, 1997a; Sirohi et al, 1999b).

Because the correct interpretation of corrected GFR values depend on patient age and body surface area, GFR was treated both as a dichotomous value (normal/abnormal dichotomy) and a continuous variable in our analysis of the data set. The analyses were undertaken in March 2000.

### RESULTS

### Patient characteristics

The patient demography at presentation and at the time of high-dose therapy is described in Tables 1 and 2. Patients with a low GFR had a higher $\beta_2$M ($P$ = < 0.0001), calcium ($P = 0.009$) and creatinine ($P = 0.0001$) and more commonly showed Durie–Salmon stage IIIA/B disease ($P < 0.0001$) and Bence–Jones proteinuria ($P = 0.002$) at presentation. At the time of high-dose, they had a significantly higher $\beta_2$M ($P = 0.02$), creatinine ($P = 0.0006$) and stage IIIA/B disease ($P = 0.0002$).

### Induction treatment

The median number of courses of IC was 5 (range, 1–9). There was no difference in the type of IC received by the low and normal GFR group but patients in the low GFR group received significantly more courses ($P = 0.001$, Table 2).

### GFR

The median GFR in the whole group of 145 patients was 87 (range, 24–140) ml min$^{-1}$ 1.73 m$^{-2}$. 38 (26.2%) patients had a GFR

**Table 1** Patient characteristics at presentation

| | **Normal GFR** ***n* = 107 (%)** | **Low GFR** ***n* = 38 (%)** | ***P* value[a]** |
|---|---|---|---|
| Age–median 51 years (range, 30–72) | | | |
| < 51 | 51 (48) | 18 (47) | 0.98 |
| ≥ 51 | 56 (52) | 20 (53) | |
| Sex | | | |
| Male | 71 (66) | 25 (66) | 0.9 |
| Female | 36 (34) | 13 (34) | |
| Subtype | | | |
| IgG | 66 (62) | 23 (60) | |
| IgA | 13 (12) | 6 (16) | |
| IgM | 3 (3) | 0 | |
| IgD | 2 (1) | 0 | 0.8 |
| BJ | 18 (17) | 8 (21) | |
| NS | 5 (5) | 1 (3) | |
| Light chain | | | |
| kappa | 62 (58) | 27 (71) | 0.16 |
| lambda | 40 (37) | 10 (26) | |
| Bence–Jones | | | |
| Positive | 52 (49) | 30 (79) | 0.001 |
| Negative | 55 (51) | 8 (21) | |
| Calcium – median 2.39 mmol l$^{-1}$ (range, 1.75–4.26) | | | |
| < 2.39 | 60 (56) | 12 (32) | 0.009 |
| ≥ 2.39 | 47 (44) | 26 (68) | |
| $\beta_2$M – median 2.9 mg dl$^{-1}$ (range, 0.1–48.6) | | | |
| < 2.9 | 65 (61) | 6 (16) | <0.0001 |
| ≥ 2.9 | 42 (39) | 32 (84) | |
| Albumin – median 36 mmol l$^{-1}$ (range, 16–51) | | | |
| < 36 | 49 (46) | 22 (58) | 0.20 |
| ≥ 36 | 58 (54) | 16 (42) | |
| Creatinine median 87 μmol l$^{-1}$ (range, 56–572) | | | |
| <87 | 63 (59) | 8 (21) | 0.0001 |
| ≥87 | 44 (81) | 30 (79) | |
| Dure–Salmon stage | | | |
| IA/IIA | 38 (36) | 2 (5) | <0.0001 |
| IIIA/B | 64/5 (64) | 25/11 (95) | |

[a]Chi square without Yates correction

**Table 2** Patient characteristics at the time of HDM 200 mg/m²

| | Normal GFR *n* = 107 (%) | Low GFR *n* = 38 (%) | *P* value[a] | Optimum value[b] | Number (%) *n* = 145 |
|---|---|---|---|---|---|
| Median age 52 years, (range, 31–72) | | | | | |
| <52 | 51 (48) | 18 (47) | 0.96 | <56 | 97 (67) |
| ≥ 52 | 56 (52) | 20 (53) | | ≥56 | 48 (33) |
| Median calcium 2.3 mmol $l^{-1}$ (range, 1.66–2.73) | | | | | |
| <2.3 | 51 (48) | 18 (47) | 0.98 | Same as median | |
| ≥2.3 | 56 (52) | 20 (53) | | | |
| Median $\beta_2$ M 2.4 mg $dl^{-1}$ (range, 1–8) | | | | | |
| <2.4 | 58 (54) | 12 (32) | 0.023 | <1.8 | 31 (21) |
| ≥2.4 | 47 (44) | 24 (63) | | 1.8–3.4 | 92 (64) |
| | | | | >3.4 | 18 (12) |
| Missing values 2 (2) | 2 (5) | | | | 4 (3) |
| Median albumin 41 mmol $l^{-1}$ (range 26–100) | | | | | |
| <41 | 45 (42) | 17 (45) | 0.77 | ≤34 | 18 (13) |
| ≥41 | 62 (58) | 21 (55) | | >34 | 127 (87) |
| Median creatinine 78 µmol $l^{-1}$ (range, 50–182) | | | | | |
| <78 | 60 (56) | 9 (23) | 0.0006 | Same as median | |
| ≥78 | 47 (44) | 29 (77) | | | |
| Stage | | | | | |
| IA/IIA | 38 (36) | 2 (5) | 0.0002 | | |
| IIIA/IIIB | 68/1 (64) | 36/0 (95) | | | |
| HDT | | | | | |
| Marrow | 52 (49) | 18 (47) | 0.86 | | |
| PBSC | 55 (51) | 20 (53) | | | |
| VAMP type | | | | | |
| VAMP | 20 (19) | 7 (18) | 0.95 | | |
| CVAMP[c] | 86 (80) | 31 (82) | | | |
| None | 1(1) | | | | |
| Courses of IC | | | | | |
| <5 | 41 (38) | 4 (11) | 0.001 | | |
| ≥5 | 65 (61) | 34 (89) | | | |
| Median GFR 87 ml/min/m² (range, 24–140) | | | | | |
| <87 | 33 (31) | 38 (100) | | < 80 | 52 (36) |
| ≥87 | 74 (69) | 0 | | 80–100 | 53 (37) |
| | | | | >100 | 27 (27) |

[a]2 × 2 Chi squared test without Yates correction. [b]Optimum cut-off values obtained after recursive partitioning as described in the text. [c]Also includes VCVAMP.

below the normal range just prior to HDM. 3 patients had GFR < 40 ml $min^{-1}$ $m^{-2}$ (20, 24 and 38 ml $min^{-1}$ $m^{-2}$).

### Transplant-related toxicity

### *Haematological recovery*

The median number of days to neutrophil recovery above $0.5 \times 10^9$ $l^{-1}$ was the same in patients with low and normal GFR (20 days, $P = 0.7$). Recovery of platelets to > $50 \times 10^9$ $l^{-1}$ was also comparable between the groups, patients with a low GFR requiring a median of 27 days and those with a normal GFR a median of 29 days ($P = 0.89$).

### *Organ dysfunction*

Table 3 summarizes the major transplant-related toxicities. Most patients experienced some degree of mucositis, diarrhoea, nausea, vomiting and fever. The incidence of oral mucositis ($P < 0.0001$) and diarrhoea ($P = 0.005$) was significantly higher in patients with low GFR. Hepatotoxicity was negligible in both groups. Grade III/IV infections were significantly more frequent in the patients with low GFR ($P = 0.04$). The incidence of serum creatinine > 177 µmol $l^{-1}$ (equivalent to the cut-off used to define Durie–Salmon stage B) during the first 60 days after HDM was 11% in patients with a low GFR pre HDM vs. 7% in patients with a normal GFR ($P = 0.43$).

### *Period of hospitalization*

The median number of days to discharge in patients with a normal GFR was 23 days compared to 22 days in patients with a low GFR ($P = 0.82$). None of the patients required dialysis during their transplant.

### *Transplant-related mortality*

6 (4%) patients died during the autograft, 2 with low GFR (one died of pseudomonal and staphylococcal septicaemia, the other of pulmonary aspergillosis) and 4 with normal GFR group (3 neutropenic sepsis and 1 veno-occlusive disease; $P = 0.69$).

### Response

The response after IC and HDM is shown in Table 4. Before HDM, 29 (20%) patients were in CR, 92 (63%) in PR and 24 (17%) were NR. All 29 patients in CR prior to HDM continued to be in CR after HD. A further 55 (38%) went into CR during HDM

© 2001 Cancer Research Campaign

**Table 3** Transplant-related toxicity

| | Normal *n* = 107(%) | Low *n* = 38(%) | *P* value[a] |
|---|---|---|---|
| Mucositis | | | |
| Grade 0/I/II | 82 (77) | 11 (29) | <0.0001 |
| III/IV | 25 (23) | 27 (71) | |
| Diarrhea | | | |
| Grade 0/I/II | 90 (84) | 23 (61) | 0.003 |
| III/IV | 17 (16) | 15 (39) | |
| Creatinine > 177 | | | |
| Yes | 7 (7) | 4 (11) | 0.43 |
| No | 100 (93) | 34 (89) | |
| Bilirubin | | | |
| Grade 0 | 76 (71) | 32 | 0.24 |
| I/II | 29 (27) | 6 | |
| III/IV | 2 (2) | 0 | |
| Transaminases | | | |
| Grade 0 | 97 (91) | 31 (82) | 0.21 |
| I/II | 9 (8) | 7 (18) | |
| III/IV | 1 (1) | 0 | |
| Alkaline phosphatase | | | |
| Grade 0 | 83 (77) | 21 (55) | 0.01 |
| I/II | 23 (22) | 14 (37) | |
| III/IV | 1 (1) | 3 (8) | |
| Infection | | | |
| Grade 0/I | 6 (5) | 0 | 0.01 |
| II | 79 (74) | 20 (53) | |
| III | 18 (17) | 15 (39) | |
| IV | 4 (4) | 3 (8) | |

[a]Chi square without Yates correction.

**Table 4** Response to IC and HDM 200 mg $m^{-2}$

| | Normal GFR *n* = 107 (%) | Low GFR *n* = 38 (%) | *P* value |
|---|---|---|---|
| Response to IC | | | |
| CR | 22 (20) | 7 (18) | |
| PR | 66 (62) | 24 (64) | 0.8[b] |
| NR[a] | 19 (18) | 7 (18) | |
| Response to HDM | | | |
| CR+CCR | 43+22 (61) | 12+7 (50) | 0.19[b] |
| PR+CPR | 5+29 (31) | 5+12 (45) | |
| NR | 4 (4) | 0 | |
| TRM | 4 (4%) | 2 (5%) | 0.69[c] |

[a] One patient who did not receive IC is taken as a non-responder. [b] Chi-square without Yates correction. [c] Kaplan–Meier. CR=complete remission; CCR=continued complete remission; PR=partial remission; CPR=continued partial remission; NR=non responder; TRM=treatment-related mortality.

so that the total CR rate after HDM was 58% (84/145). The disease status after HDT was not significantly different between the groups.

### Survival and relapse

The median OS was 5.1 years (95% CI 2.5 to 6.2) for patients with a low GFR vs. 7.5 years (95% CI 5.5 to 12.2) in patients with a normal GFR ($P$ = 0.015, Figure 1) and the median EFS was 2.1 years (95% CI 1.1 to 3.9) for patients with a low GFR vs. 2.6 years (95% CI 2.1 to 3.7) for patients with a normal GFR ($P$ = 0.24, Figure 2).

There was no statistically significant difference in OS ($P$ = 0.47) and EFS ($P$ = 0.08) in patients receiving either autologous bone marrow or peripheral stem cell rescue.

In the low GFR group, 22/38 patients (58%) relapsed at a median of 2.8 years (range, 0.2 to 5.9), compared to 53/107 (49%) at a median of 4.1 years (range, 0.4 to 7.1, $P$ = 0.16) in the group with a normal GFR. Importantly, the median OS after relapse in the 22 patients with a low GFR at the time of HDT was only 0.85 years (range, 0.02 to 5.5) compared to 3.4 years (range, 0.14 to 6.7) in the 53 patients with a normal GFR ($P$ = 0.02, Figure 3).

The median serum creatinine of patients in the low GFR group at relapse was 96 (range, 58–141) compared to 82 (range, 52–728) in the low GFR group ($P$ = 0.004), and there was no significant difference in the intensity of treatment given to these patients after relapse between the 2 groups. 31/53 (59%) in the normal GFR group compared to 9/22 (41%) in the low GFR group received infusional chemotherapy followed by a second high-dose therapy ($P$ = 0.16). At the time of second high-dose therapy, 5/31 (16%) vs. 4/9 (44%) respectively had a low GFR ($P$ = 0.07).

The multivariate analysis was carried out using partitioning in the way described above. To be consistent with our previously published data and to have an even distribution of patients, we have used medians for the continuous variables and the categorical variables were taken as described above.

(a) Patients with a normal GFR and in CR before HDM had a significantly longer OS and achieving CR before HDM was also predictive of longer EFS (Table 5a)
(b) Younger patients (age < 56 years) with a low $\beta_2$M (< 1.8 better than 1.8–3.4 which in turn is better than > 3.4) had prolonged OS while those with a higher albumin (> 34) and a better response prior to HDM (CR better than PR which is better than NR) had a significantly prolonged EFS (Table 5b).

Recursive partitioning showed what appeared to be a strong influence of values away from the median. This is likely to be spurious since at cut-off points away from median, one arm in the Kaplan–Meier plot may contain only few patients. In such small samples, the existence of atypical patients in one arm may produce a large but atypical effect, but a large number of events in these groups will have prognostic significance.

### DISCUSSION

The staging of myeloma patients with respect to their degree of renal impairment is commonly based on serum creatinine measurements (Durie and Salmon, 1975). However, serum creatinine levels do not increase unless a significant drop in GFR has already occurred and GFR is considered to be more accurate as a measure of renal function (Britton et al, 1979; Labeeuw et al, 1994; Kasiska and Keane, 1996; Toto et al, 1997). The clinical data available in the literature relating to high-dose chemotherapy in myeloma patients with renal impairment is limited. In the past, myeloma patients with diminished renal function in our unit received high-dose busulphan (Mansi et al, 1991). However, patients receiving high-dose busulphan as well as those receiving high-dose melphalan at 140 mg $m^{-2}$ had a significantly worse progression-free ($P$ = 0.0003) and overall ($P$ < 0.0001) survival compared with those receiving melphalan 200 mg $m^{-2}$ (Powles

© 2001 Cancer Research Campaign 

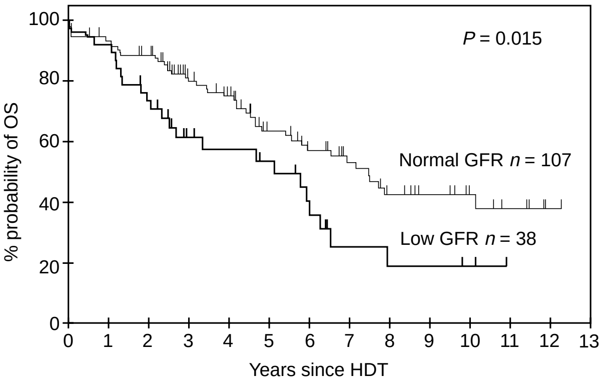

**Figure 1** Log rank comparison of overall survival (OS) of patients with normal GFR ($n$ = 107) and low GFR ($n$ = 38) post high-dose therapy (HDT). $P$ = 0.015

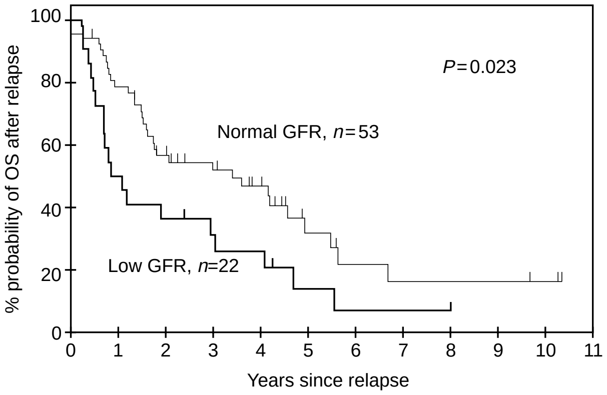

**Figure 3** Log-rank comparison of overall survival (OS) of patients who relapsed after autograft in normal GFR ($n$ = 53) and low GFR ($n$ = 22) group; $P$ = 0.023

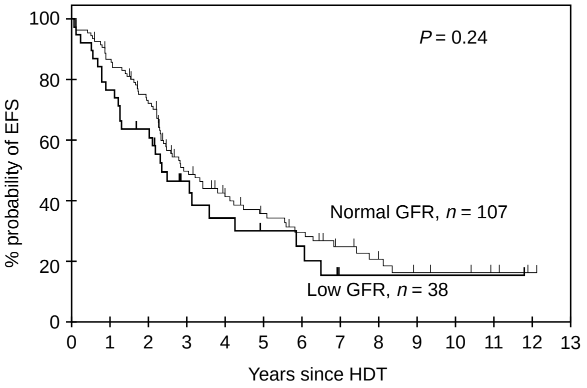

**Figure 2** Log-rank comparison of event-free survival (EFS) of patients with normal ($n$ = 107) and low GFR ($n$ = 38) post high-dose therapy (HDT); $P$ = 0.24

et al, 1997). This was also demonstrated in a multicentre EBMT study of autografting in myeloma in which conditioning regimens that contained HDM were associated with significantly longer progression-free and overall survival rates (Bjorkstrand et al, 1994). Presently, HDM 200 mg $m^{-2}$ is probably the most commonly used regimen in patients with multiple myeloma (Bjorkstrand et al, 1994; Powles et al, 1997; Barlogie et al, 1999) and studies with the escalation of the melphalan dose to 220 mg $m^{-2}$ are currently being undertaken (Moreau et al, 2000). In 1994, Kergueris et al reported that adjusting the dose of melphalan according to renal function was not warranted. Because of the proven value of HDM in the treatment of myeloma we have routinely treated myeloma patients with abnormal renal function with HDM 200 mg $m^{-2}$ according to a care plan approved by the institutional ethics committee. Here we set out to assess the tolerability and efficacy of this therapeutic strategy.

Only 3 patients had a GFR < 40 ml $min^{-1}$ $m^{-2}$. There was no difference in neutrophil and platelet recovery between patients with low or normal GFR. While the potentially increased myelotoxicity of HDM in patients with low GFR may have been masked by the stem cell rescue, the presence of renal impairment did not cause any apparent cytotoxic damage to the autograft by the HDM given 24 hours earlier. This is in keeping with the predominantly non-renal clearance of melphalan (Kergueris et al, 1994; Tricot et al, 1996)

Interestingly, however, despite the independence of the area under the curve (AUC) of melphalan from the GFR, we observed significantly more mucosal toxicity after HDM in patients with a low GFR. In their group of 20 patients Tricot et al (1996) observed that there was a trend towards a longer duration of mucositis in patients with a creatinine clearance less than 40 ml $min^{-1}$ who received HDM 200 mg $m^{-2}$. Severe mucositis also featured prominently in the French study (Kergueris et al, 1994). It is possible that independent of the AUC, tissue injury by melphalan is increased in the presence of uraemia.

The increased gastrointestinal toxicity of HDM in the presence of renal impairment may explain the 2.8-fold increase in the incidence and the greater severity of microbiologically documented infections among our patients with a low GFR. There may have been a higher rate of bacterial translocation from the gut into the circulation across damaged mucosa in the low GFR group. Tricot et al showed that patients with impaired renal function had a longer duration of fever ($P$ = 0.0005) compared with those with normal renal function. Nevertheless, unlike in Tricot's study, in our cohort of patients the increased risk of infection did not translate into a longer hospital stay.

The treatment-related mortality was not significantly different between the 2 groups. Thus, with good supportive care, autotransplants with HDM 200 mg $m^{-2}$ are safe whatever the renal function, but patients with a low GFR should possibly receive prophylactic antibiotics to reduce the transplant-related morbidity associated with oral mucositis, diarrhoea and infections.

In the multivariate analysis, the factor that most strongly correlated with a better event free as well as overall survival was CR immediately before transplantation, confirming previous reports by ourselves and several other workers (Selby et al, 1988; Bjorkstrand et al, 1994; Attal et al, 1996; Sirohi et al, 1999b). Given the importance of achieving CR at the time of high-dose therapy, intensification of induction treatment may be of benefit, but consolidation with high-dose intensification in these patients remains a reasonable approach as the CR rate was increased from 20% to 58% by HDM. Since patients with low GFR required a higher number of courses of IC to attain maximum response it appears that they presented with more chemo-resistant disease.

However, despite the safety of HDM in myeloma patients with low GFR and their similar EFS to patients with normal GFR, the long-term outcome of low GFR patients was significantly worse as patients with a low GFR who relapsed after HDM had a significantly shorter OS. The TRM after second line treatment was not significantly different between the 2 groups (6/53 (11%) died in

 *© 2001 Cancer Research Campaign*

**Table 5a** Multivariate analysis for outcome in 145 patients who received HDT (medians of continuous variables were used and GFR was categorized as Normal or Low according to the definition in the text)

| Event | Variables at the time of HDM | Relative risk | 95% CI | *P* value |
|---|---|---|---|---|
| OS | GFR | 0.53[a] | 0.31–0.91 | 0.02 |
| | Disease status at HDM | 0.63 | 0.41–0.91 | 0.02 |
| EFS | Disease status at HDM* | 0.59[b] | 0.41–0.83 | 0.002 |

[a] Patients with normal GR at the time of HDM were only 53% likely to die following HDM than patients with low GFR. [b] Patients achieving CR to IC were only 59% as likely to experience death or relapse following HDM than patients who did not achieve CR with IC.

**Table 5b** Multivariate analysis for outcome in 145 patients who received HDT (the continuous variables had an optimum cut-off chosen by recursive partitioning)

| Event | Variables at the time of HDM | Relative risk | 95% CI | *P* value |
|---|---|---|---|---|
| OS | Age | 0.48 | 0.27–0.85 | 0.001 |
| | $\beta_2$M | 0.55 | 0.34–0.89 | 0.01 |
| EFS | Albumin | 0.31 | 0.15–0.65 | 0.002 |
| | Disease status at HDM | 0.6 | 0.41–0.89 | 0.01 |

$\beta_2$M = beta 2 microglobulin.

the group with normal GFR versus 2/22 (9%) in the low GFR group; $P = 0.78$). It is possible that myeloma patients with low GFR may benefit from more intensive treatment and supportive care at the time of relapse.

The multivariate analysis using the variables chosen by recursive partitioning have shown that previously described variables such as age, $\beta_2$M, albumin and disease status prior to HDM affected the outcome (Sirohi et al, 1999b), further underlining that the dose of melphalan should not be modified in patients with abnormal renal function.

In summary, our experience suggests that the dose of melphalan need not be reduced in patients with renal failure receiving HDM. Patients should receive chemotherapy aimed at increasing the CR rate from the outset, as this is the strongest prognostic factor for both prolonged OS and EFS. Nevertheless, patients with abnormal renal function at the time of high-dose treatment have increased transplant-related morbidity. After relapse, their prognosis is worse than that of patients whose pre-transplant GFR is normal and incisive treatment strategies with optimal supportive care are required for this patient subgroup at the time of relapse. Our conclusions will need to be confirmed in a prospective study.

### ACKNOWLEDGEMENTS

We thank the David Adams Leukaemia Fund, Institute of Cancer Research, and the Bud Flanagan Leukaemia Fund.

### REFERENCES

Attal M, Harousseau JL, Stoppa AM, Sotto JJ, Fuzibet JG, Rossi JF, Cassassus P, Maisonneuve H, Facon T, Ifrah N, Payen C and Bataille R (1996) A prospective, randomized trial of autologous bone marrow transplantation and chemotherapy in multiple myeloma. Intergroupe Francais du Myelome. *N Engl J Med* **335**: 91–97

Barlogie B, Jagannath S, Desikan KR, Mattox S, Vesole D, Siegal D, Tricot G, Munshi N, Fassas A, Singhal S, Mehta J, Anaissie E, Dhodapkar D, Naucke S, Cromer J, Sawyer J, Epstein J, Spoon D, Ayers D, Cheson B and Crowley J (1999) Total therapy with tandem transplants for newly diagnosed multiple myeloma. *Blood* **93**: 55–65

Bjorkstrand B, Goldstone AH, Ljungman P, Brandt L, Brunet S, Carlson K, Prentice HG, Cavo M, Samson D, Laurenzi AD, Verdonck LF, Proctor S, Ferrant A, Sierra J, Auzanneau G, Troussard X, Gravett P, Remes K and Gahrton G (1994) Prognostic factors in autologous stem cells transplantation for multiple myeloma: an EBMT Registry study. *Leuk Lymphoma* **15**: 265–272

BMDP statistical software release 7 (1992). BMDP Statistical Software Manual, University of California Press, Berkeley, **2**: 825–866

Britton KE, Nimmon CC, Whitfield HN, Hendry WF and Wickham JEA (1979) Obstructive nephropathy: successful evaluation with radionuclides, *Lancet*, **i**: 905

Brochner-Mortensen J (1978) Routine methods and their reliability for assessment of glomerular filtration rate in adults. *Danish Medical Bulletin* **25**(5): 80–98

Cox DR (1972) Regression models and life tables. *Journal of Royal Statistical Society of Britain* **24**: 187–220

Cunningham D, Paz-Ares L, Milan S, Powles R, Nicolson M, Hickish T, Selby P, Treleaven J, Viner C, Malpas J, Slevin M, Findlay M, Raymond J and Gore ME (1994) High dose melphalan and autologous bone marrow transplantation as consolidation in previously untreated myeloma. *J Clin Oncol* **12**: 759–736

Cunningham D, Powles RL, Malpas J, Raje N, Milan S, Viner C, Montes A, Hickish T, Nicolson M, Johnson P, Treleaven J, Raymond J and Gore M (1998) A randomised trial of maintenance interferon following high dose chemotherapy in multiple myeloma; long term follow up results. *Br J Haematol* **102**: 495–502

Durie BGM and Salmon SE (1975) A clinical staging system for multiple myeloma. *Cancer* **36**: 842–854

Forgeson GV, Selby P, Lakhani S, Sulian G, Viner C, Maitland J and McElwain TJ (1988) Infused vincristine and adriamycin with high dose methylprednisolone (VAMP) in advanced previously treated multiple myeloma patients. *Br J Cancer* **58**: 469–473

Gore ME, Selby PJ, Millar B, Maitland J and McElwain TJ (1988) The use of verapamil to overcome drug resistance in myeloma. *Proc Am Soc Clin Oncol* **7**: 228

Gore ME, Selby PJ, Viner C, Clark PI, Meldrum H, Millar B, Bell J, Maitland JA, Milan S, Judson IR, Suiable A, Tillyer C, Slevin M, Malpas JS and McElwain TJ (1989) Intensive treatment of Multiple Myeloma and criteria for complete remission. *Lancet* **2**: 879–881

Kaplan EL and Meier P (1958) Non parametric estimation from incomplete observations. *J Am Stat Assoc* **53**: 457–481

Kasiske BL and Keane WF (1996) Laboratory assessment of renal disease: Clearance, urinanalysis and renal biopsy in Brenner BM, Rector FC Jr (eds): The Kidney (ed 5). Philadelphia, PA, Saunders: 1137–1173

Kergueris MF, Milpied N, Moreau N, Harousseau JL and Larousse C (1994) Pharmacokinetics of high-dose melphalan in adults: influence of renal function. *Anticancer Res* **14**: 2379–2382

Kruskal WH and Wallis WA (1952) Use of ranks in one-criterion variance analysis, *J Am Stat Assoc* **47**: 583–621

Labeeuw M, Diaz C, Cailette A, Aissa AH and Pozet N (1994) Estimation of GFR from serum creatinine in elderly patients: comparison of several methods, *J Am Soc Nephrol* **5**: 336a

Leblanc M and Crowley J (1993) Survival trees by goodness of split. *J Am Stat Assoc* **88**: 457–467

Mansi J, Da Costa F, Viner C, Judson I, Gore M and Cunningham D (1991) High dose busulfan in patients with myeloma. *J Clin Oncol* **10**: 1569–1573

Medical Research Council Working Party on Leukemia in adults. (1980) Prognostic features in the third MRC myelomatosis trial. *British Journal of Cancer* **42**: 831–840

Merlini G, Waldenstrom JG and Jayakar S (1980) A new improved clinical staging system for multiple myeloma based on analysis of 123 treated patients. *Blood* **55**: 1011–1019

Miller AB, Hoogstraten B, Staquet M and Winkler A (1981) Reporting results of Cancer treatment, *Cancer* **47**: 207–214

Moreau P, Milpied N, Mahe B, Juge-Morineau N, Rapp M-J, Bataille R and Harousseau J-L (1999) Melphalan 220 mg/m$^2$ followed by peripheral blood stem cell transplantation in 27 patients with advanced multiple myeloma. *Bone Marrow Transplant* **23**: 1003–1006

Peto R, Pike MC, Armitage P, Breslow NE, Cox DR, Howard SV, Mantel N, McPherson K, Peto J and Smith PG (1977) Design and analysis of randomized clinical trials requiring prolonged observation of each patient. II, Analysis and examples. *Br J Cancer* **35**: 1–39

© 2001 Cancer Research Campaign 

Powles R, Raje N, Milan S, Millar B, Shepherd V, Mehta J, Singhal S, Kulkarni S, Viner C, Gore M, Cunningham D and Treleaven J (1997) Outcome assessment of a population based group of 195 unselected myeloma patients under 70 years of age offered intensive treatment. *Bone Marrow Transplant* **20**: 435–443

Raje N, Powles R, Kulkarni S, Milan S, Middleton G, Singhal S, Mehta J, Millar B, Viner C, Raymond J and Treleaven J (1997a) A comparison of vincristine and doxorubicin infusional chemotherapy with methylprednisolone (VAMP) with the addition of weekly cyclophosphamide (C-VAMP) as induction treatment followed by autografting in previously untreated myeloma. *Br J Haematol* **97**: 153–160

Raje N, Powles R, Horton C, Millar B, Shepherd V, Middleton G, Kulkarni S, Eisen T, Mehta J, Singhal S and Treleaven J (1997b) Comparison of marrow vs blood derived stem cells for autografting in previously untreated multiple myeloma. *Br J Cancer* **75**: 1684–1689

Selby P, Zulian G, Forgeson G, Nandi A, Milan S, Meldrum M, Viner C, Osborne R, Malpas JS and McElwain TJ (1988) The development of high-dose melphalan and of autologous bone marrow transplantation in the treatment of multiple myeloma: Royal Marsden and St Bartholomew's Hospital studies. *Haematol Oncol* **6**: 173–179

Siegel DS, Desikan KR, Mehta J, Singhal S, Fassas A, Munshi N, Annaissie E, Naucke S, Ayers D, Spoon D, Vesole D, Tricot G and Barlogie B (1999) Age is not a prognostic variable with autotransplants for multiple myeloma. *Blood* **93**: 51–54

Sirohi B, Powles R, Treleaven J, Singhal S, Kulkarni S, Parikh P, Bhagwati N, Horton C and Mehta J (1999a) The implication of compromised renal function at the time of presentation in myeloma: a single centre study of 251 patients. *Blood* **94**: Suppl 1, 575a

Sirohi B, Powles R, Mehta J, Raje N, Kulkarni S, Ramiah V, Saso R, Horton C, Bhagwati N, Singhal S and Treleaven J (1999b) Complete remission rate and outcome after intensive treatment of 177 patients under 75 years of age with IgG myeloma defining a circumscribed disease entity with a new staging system. *Br J Haematol* **107**: 656–666

Sirohi B, Powles R, Treleaven J, Mainwaring P, Kulkarni S, Pandha H, Bhagwati N, Horton C, Singhal S and Mehta J (2000) The role of autologous transplantation in patients with multiple myeloma aged 65 years and over. *Bone Marrow Transplant* **25**: 533–539

Toto RD, Kirk KA, Coresh J, Jones C, Appel L, Wright J, Campese V, Oltunde B, Agodoa L, and AASK Pilot Study Investigators (1997) Evaluation of serum creatinine for estimating glomerular filtration rate in blacks with hypertensive nephrosclerosis: Results from the African-American Study of Kidney disease and hypertension (AASK) Pilot study. *J Am Soc Nephrol* **8**: 279–287

Tricot G, Alberts DS, Johnson C, Roe DJ, Dorr RT, Bracy D, Vesole DH, Jagannath S, Meyers R and Barlogie B (1996) Safety of autotransplants with high-dose melphalan in renal failure: a pharmacokinetic and toxicity study. *Clin Cancer Res* **2**: 947–952

Vesole DH, Tricot G, Jagannath S, Desikan KR, Siegel D, Bracy D, Langdon M, Cheson B, Crowley J and Barlogie B (1996) Autotransplants in multiple myeloma: what have we learned? *Blood* **88**: 838–847

*© 2001 Cancer Research Campaign*
